# Pediatric and Young Adult Household Transmission of the Initial Waves of SARS-CoV-2 in the United States: Administrative Claims Study

**DOI:** 10.2196/44249

**Published:** 2024-01-04

**Authors:** Ming Kei Chung, Brian Hart, Mauricio Santillana, Chirag J Patel

**Affiliations:** 1 Department of Biomedical Informatics Harvard Medical School Harvard University Boston, MA United States; 2 Jockey Club School of Public Health and Primary Care The Chinese University of Hong Kong Hong Kong China (Hong Kong); 3 Institute of Environment, Energy, and Sustainability The Chinese University of Hong Kong Hong Kong China (Hong Kong); 4 Optum Labs Eden Prairie, MN United States; 5 Machine Intelligence Group for the Betterment of Health and the Environment Network Science Institute Northeastern University Boston, MA United States

**Keywords:** household transmission, infectivity, pediatric, COVID-19, children, claims data, administrative data, transmission, risk factor, logistic regression, regression, multivariable logistics regression, multiple logistic regression statistics, cohort, retrospective cohort, laboratory, LOINC, infant, toddler, newborn

## Abstract

**Background:**

The correlates responsible for the temporal changes of intrahousehold SARS-CoV-2 transmission in the United States have been understudied mainly due to a lack of available surveillance data. Specifically, early analyses of SARS-CoV-2 household secondary attack rates (SARs) were small in sample size and conducted cross-sectionally at single time points. From these limited data, it has been difficult to assess the role that different risk factors have had on intrahousehold disease transmission in different stages of the ongoing COVID-19 pandemic, particularly in children and youth.

**Objective:**

This study aimed to estimate the transmission dynamic and infectivity of SARS-CoV-2 among pediatric and young adult index cases (age 0 to 25 years) in the United States through the initial waves of the pandemic.

**Methods:**

Using administrative claims, we analyzed 19 million SARS-CoV-2 test records between January 2020 and February 2021. We identified 36,241 households with pediatric index cases and calculated household SARs utilizing complete case information. Using a retrospective cohort design, we estimated the household SARS-CoV-2 transmission between 4 index age groups (0 to 4 years, 5 to 11 years, 12 to 17 years, and 18 to 25 years) while adjusting for sex, family size, quarter of first SARS-CoV-2 positive record, and residential regions of the index cases.

**Results:**

After filtering all household records for greater than one member in a household and missing information, only 36,241 (0.85%) of 4,270,130 households with a pediatric case remained in the analysis. Index cases aged between 0 and 17 years were a minority of the total index cases (n=11,484, 11%). The overall SAR of SARS-CoV-2 was 23.04% (95% CI 21.88-24.19). As a comparison, the SAR for all ages (0 to 65+ years) was 32.4% (95% CI 32.1-32.8), higher than the SAR for the population between 0 and 25 years of age. The highest SAR of 38.3% was observed in April 2020 (95% CI 31.6-45), while the lowest SAR of 15.6% was observed in September 2020 (95% CI 13.9-17.3). It consistently decreased from 32% to 21.1% as the age of index groups increased. In a multiple logistic regression analysis, we found that the youngest pediatric age group (0 to 4 years) had 1.69 times (95% CI 1.42-2.00) the odds of SARS-CoV-2 transmission to any family members when compared with the oldest group (18 to 25 years). Family size was significantly associated with household viral transmission (odds ratio 2.66, 95% CI 2.58-2.74).

**Conclusions:**

Using retrospective claims data, the pediatric index transmission of SARS-CoV-2 during the initial waves of the COVID-19 pandemic in the United States was associated with location and family characteristics. Pediatric SAR (0 to 25 years) was less than the SAR for all age other groups. Less than 1% (n=36,241) of all household data were retained in the retrospective study for complete case analysis, perhaps biasing our findings. We have provided measures of baseline household pediatric transmission for tracking and comparing the infectivity of later SARS-CoV-2 variants.

## Introduction

Since the outbreak of COVID-19 in early 2020, over 500 million cases and 6 million deaths have been recorded by the World Health Organization [1]. In the United States alone, over 81 million people have been infected, and almost 1 million people have died due to SARS-CoV-2 [1]. Characterizing the transmission dynamics of the evolving virus provides important evidence to assist in formulating effective public health interventions to fight the pandemic. For example, recent research utilizing data from over 80, 000 household contacts estimated the infectivity of the Omicron and Delta variants of SARS-CoV-2 [2].

The Centers for Disease Control and Prevention (CDC) is responsible for keeping track of the COVID-19 pandemic and providing vital national statistics about COVID-19 in the United States, including new cases, deaths, hospitalizations, and vaccination rates [3]. However, location and family-related information is typically not collected; thus, it is difficult to estimate infectivity, such as the household secondary attack rate (SAR) and odds of SARS-CoV-2 transmission to family members. In European countries, such as England and Denmark, it is possible to link individual SARS-CoV-2 test results to the national administrative records in centralized health care systems and registers and thereby obtain address information [4-6]. Currently, the National COVID Cohort Collaborative is one of the largest United States counterparts with data from over 13 million patients for COVID research [7]. Nevertheless, because of privacy regulations, location information is not readily accessible for estimating the infectivity of SARS-CoV-2 to family members.

There are limited reports on household transmission of SARS-CoV-2 in the United States, especially during the first year of the COVID-19 pandemic. Most of the studies were small in sample size (between 100 and 400), sampled in a particular state, and spanned 1 month to a few months [8-11]. Therefore, the estimated transmission rate of SARS-CoV-2 had a large variation with reported values of SARs between 25% and 38%, and the source of the variation is a challenge to pinpoint. In addition, the relationship between transmission risk and family size has been inconsistent [4,12-14]. A few studies compared the SARs between young adult (age 18 years or older) and child (age below 18 years) contacts and reported that adults were generally more susceptible to infection (between 28% and 48%) than children (between 16% and 28%) [8-10]. Currently, little is known about the relative infectivity of children and young adult index cases in a household setting in the United States [12,13]. Therefore, a full understanding of the baseline transmissibility in the initial waves of SARS-CoV-2 (ie, pre–SARS-CoV-2 Delta variant) in the United States can fill an important knowledge gap. This understanding serves as a crucial complement, enabling informed assessments of the infectivity and effectiveness of vaccination against other variants of concern, such as Omicron and Delta [15].

In this study, we hypothesized that the pediatric household transmission of SARS-CoV-2 in the United States was higher in the first outbreak than in the subsequent outbreaks. We aimed to repurpose administrative data to estimate pediatric household transmission of SARS-CoV-2 in the United States between January 2020 and February 2021, a critical time in the trajectory of the pandemic. We leveraged a large nationwide administrative claims data source and estimated the temporal trend of SARs. We also compared the relative infectivity (as the odds ratios [ORs] of SARS-CoV-2 transmission) between 4 pediatric index case age groups (0 to 4 years, 5 to 11 years, 12 to 17 years, and 18 to 25 years) while adjusting for other factors such as family size, quarter of infection, and residential regions to understand the early SARS-CoV-2 transmission dynamics.

## Methods

### Data Source

We conducted a retrospective cohort analysis on the household transmission of SARS-CoV-2 using deidentified administrative claims. The data source contained comprehensive inpatient and outpatient claims records, laboratory test results, and individual and various aggregated demographic information. In this study, we leveraged the outpatient laboratory data set for SARS-CoV-2 to assemble the cohort.

### Ethical Considerations

The Harvard Medical School Institutional Review Board waived the approval requirement for this study as our database analysis is not regarded as human participants research (IRB20-0935).

### Study Population

#### Identification of Households

We identified familial relationships through a family identification variable available in the demographic data table. Because of the nature of the commercial administrative data, only residential private households were included. Since household identification was based on the relationships between employee subscribers and the beneficiary members, most of the households were single- or 2-generation families.

#### SARS-CoV-2 Infection

We used the Logical Observation Identifiers Names and Codes (LOINC) information submitted by the laboratory vendors to populate the SARS-CoV-2 records, including test dates, types, and results in the outpatient laboratory data set. An individual could be tested at multiple time points with different test results. We defined a SARS-CoV-2 case as an individual tested positive with polymerase chain reaction (PCR) assay. Among the 19,028,401 rows of records, over 78.30% (n=14,899,238) were PCR tested and 10.22% (n=1,522,923) were positive.

#### Outcomes

We used SAR to measure household transmission of SARS-CoV-2, which can be interpreted as the probability of new infection among susceptible household members caused by a single case. We defined pediatric index cases (children and young adults) as those aged between 0 and 25 years who had the earliest record of SARS-CoV-2 positives in the households. We defined household contacts as all the individuals within the same household excluding the index cases. For secondary cases, we classified those individuals with SARS-CoV-2 positive 2 to 14 days after the index cases [4].

#### Assembling the Cohort

**Figure 1:**
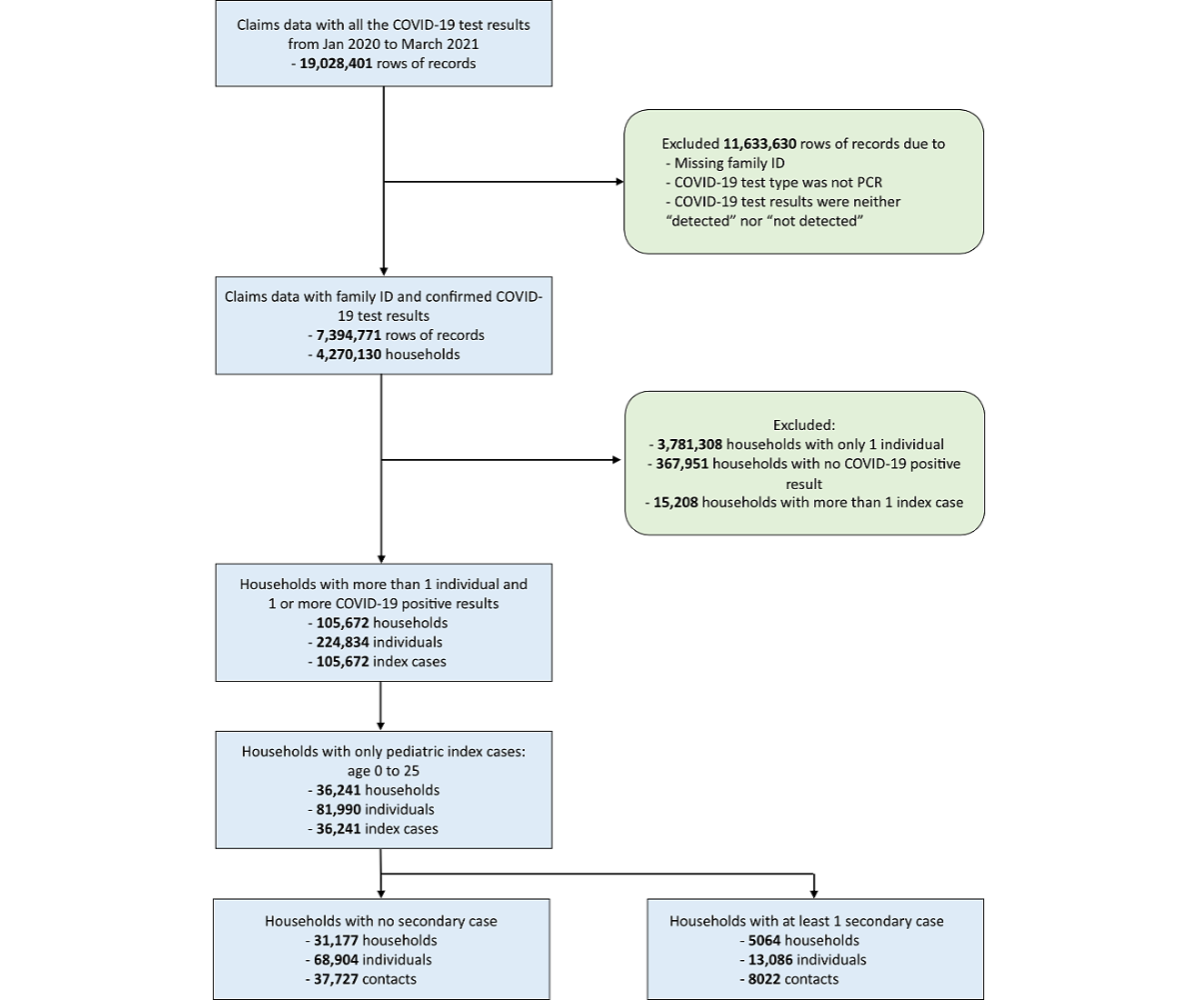
Workflow to filter administrative claims records and assemble the study cohort. PCR: polymerase chain reaction.

We created the analysis cohort as described in Figure 1. We first started with all records tested between January 1, 2020, and February 28, 2021, in the laboratory data set because we were only able to obtain complete data within this period at the time of the analysis. We did not restrict subscribers to have continuous enrollment in their health plans because the window of our analysis was short (2 weeks). There were 19,028,401 rows of records, of which we excluded 11,633,630 (61.14%) mostly because of missing family information to identify the corresponding household members. We tentatively identified 4,270,130 households and further excluded (1) 3,781,308 (88.55%) households because only a single member could be found, (2) 367,961 (8.62%) households because of missing SARS-CoV-2 cases, and (3) 15,208 (0.36%) households with more than 1 index case to avoid complicating the estimation of the relationship between index case and household transmission. After including only households with pediatric index cases, we were able to retain 36,241 effective households for the analysis (0.85% of the 4,270,130 households). We found that 31,177 (86.02%) of the households had no secondary case and 5,064 (13.97%) had at least 1 secondary case.

#### Study Variables

We derived the age variable based on the date of birth information in the administrative claims. We derived the month and quarter of the year of SARS-CoV-2 positive variables based on the date of the laboratory test records. Next, we estimated the family size in each household by counting individuals with the same family identification number. We categorized the date of the first SARS-CoV-2 positive into 5 quarters (between 2020 to 2021). Finally, we used the Census Bureau regions and divisions classification and the residential state information to create the regional division variable with 10 categories.

### Statistical Analysis

We calculated SAR as the total number of secondary cases divided by the total number of household contacts in the population. To estimate monthly SARs, we grouped households based on the monthly SARS-CoV-2 test records of the pediatric index case (ie, the first to the last day of the corresponding months). We estimated the standard errors by bootstrapping and calculated the corresponding 95% CIs of the mean SAR.

To understand the dynamic of household transmissions in the pediatric population, we conducted a stratified analysis to estimate the SARs in the age groups of pediatric index cases. Specifically, we created 4 pediatric age groups (0 to 4 years, 5 to 11 years, 12 to 17 years, and 18 to 25 years) and calculated the mean SARs and 95% CIs as described previously.

We conducted a descriptive analysis of the pediatric index cases by the 4 age groups. To quantify the associations between pediatric index case age groups and the household transmission of SARS-CoV-2 to contacts, we used logistic multiple regression to obtain the ORs using the oldest pediatric group (18 to 25 years) as the reference. We modeled the outcome as a binary variable of whether the index case is linked with any secondary cases in the corresponding household. We ran three different regression models to gauge the robustness of our analysis [16]: (1) a crude model with only the pediatric index case age groups as the predictors; (2) an adjusted model that accounted for the influences of sex, season of SARS-CoV-2 test of the pediatric index cases, and family size; and (3) a model further adjusted with the residential region of the pediatric index cases. We set a 2-sided statistical significance level of *P*=.05 for all the analyses. We executed our analyses using R software (version 4.0.2; R Foundation for Statistical Computing) and used the *boot* package for the bootstrapping step. 

## Results

We started with over 19 million SARS-CoV-2 test records, which were collected in the first 16 months of the COVID-19 pandemic in the United States We filtered the records and identified 36, 241 eligible households with pediatric index cases for the study. Most of these households (n=31,177, 86.02%) did not report any secondary cases (Figure 1).

We outline the major demographic and associated characteristics of the index case age groups in Table 1. The number of individuals in each age group is correlated with the order of the age groups. The oldest age group (18 to 25 years) had 21.9 times the individuals found in the youngest one (0 to 4 years). In each age category, we found roughly equal numbers of female and male individuals. However, the difference was larger in the oldest age group, which comprised 54.1% (n=13,394) female individuals versus 45.9% (n=11,363) male individuals. The majority of the SARS-CoV-2 cases were detected in the fourth quarter of 2020, followed by the first quarter of 2021. Over half the index cases were found in these 2 quarters. The median household size was 2 across all the age groups. The number of index cases varied greatly across 10 regional divisions, from around 2% in the East South-Central states (Alabama, Kentucky, Mississippi, and Tennessee) to 25% in the South Atlantic states (Delaware, Florida, Georgia, Maryland, North Carolina, South Carolina, Virginia, Washington, DC, and West Virginia).

**Figure 2:**
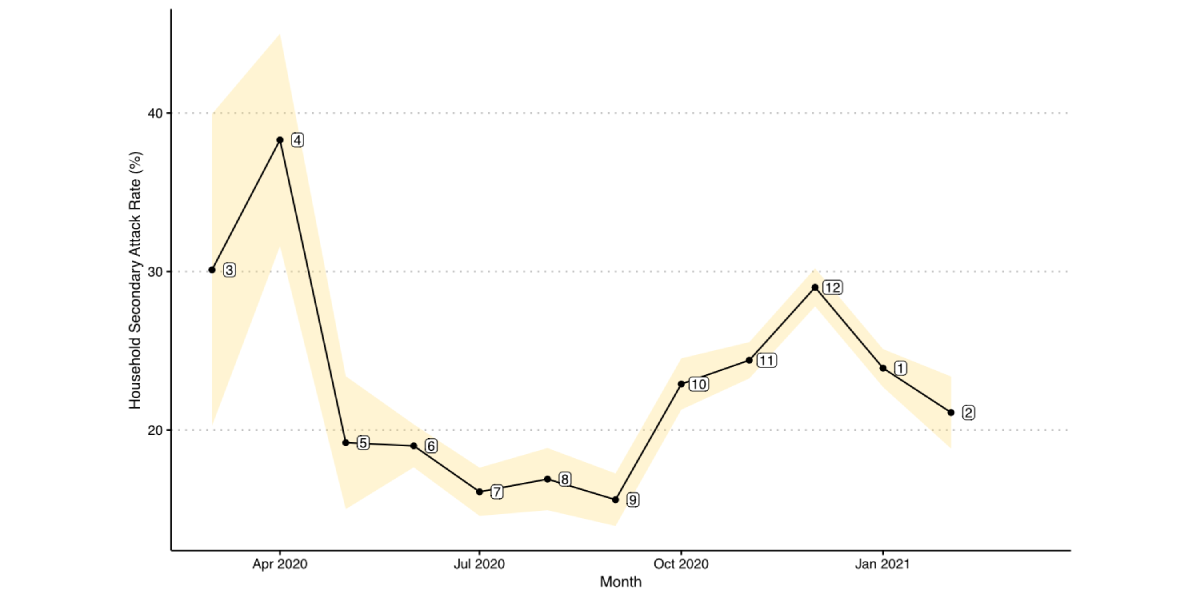
Monthly household secondary attack rates of SARS-CoV-2 for the pediatric index cases in the United States between March 2020 and February 2021. The shaded area represents the 95% CI for each month.

We plot the temporal changes of monthly SARs in Figure 2. Detailed numeric values for the graph can be found in Multimedia Appendix 1. Although our data source contained SARS-CoV-2 test records from January 1, 2020, to March 2021, we could only identify valid households between March 2020 and February 2021. The overall SAR in this period was 23.04% (95% CI 21.88-24.19). Monthly SARs varied from the lowest in September 2020 at 15.6% (95% CI 13.9-17.3) to the highest in April 2020 at 38.3% (95% CI 31.6-45). We observed 3 distinctive trends in the studied period, in that the SAR first generally decreased from April to September 2020, followed by a rebound to a peak SAR of 29% (95% CI 27.8-30.2) in December 2020 before starting to drop again until February 2021.

We show the household transmission rate of SARS-CoV-2 in the pediatric index case age groups in Multimedia Appendix 2. We found a decreasing trend of household transmission, from a SAR of 32.0% to 21.1%, as the age of the index groups increased. Specifically, the SARs were 24.5% for the 12 to 17 years age group (95% CI 23.4-25.6) and 21.1% for the 18 to 25 years age group (95% CI 20.5-21.7). These percentages were significantly lower compared to the SAR of 32.0% for the age group of 0 to 4 years (95% CI 28.8-35.2)

We presented the logistic regression results of the associations between household transmission and pediatric age groups in Table 2. First, we compared the absolute and percentage counts of index cases with and without linkage to any secondary cases by each of the predictors. All the predictors had less than 5% differences between 2 groups other than “2020 third quarter,” “2020 fourth quarter,” and “0-4 years” index age. Our analyses showed that the relationships between the age of the pediatric index cases and household SARS-CoV-2 transmission were consistent across all the investigated models—the younger the index cases were, the higher the odds of transmission. In model 2, we adjusted for all the available relevant predictors and found that the youngest index case group (0 to 4 years) had 1.69 times the odds of transmitting the SARS-CoV-2 to other household contacts (95% CI 1.42-2.00) compared to the oldest reference group (0 to 18 years).

**Table 1:** Characteristics of the index cases by age groups (N=36,241).

Characteristics of index cases			Age groups						
			0-4 years (n=1132)		5-11 years (n=3378)		12-17 years (n=6974)		18-25 years (n=24,757)
Age (years), median (IQR)			3 (2)		8 (4)		15 (3)		21 (3)
**Sex, n (%)**									
	Female	550 (48.6)		1642 (48.6)		3480 (49.9)		13,394 (54.1)	
	Male	582 (51.4)		1736 (51.4)		3494 (50.1)		11,363 (45.9)	
**Quarter of first SARS-CoV-2 positive, n (%)**									
	2020 first quarter	4 (0.4)		5 (0.1)		11 (0.2)		237 (1)	
	2020 second quarter	70 (6.2)		182 (5.4)		326 (4.7)		3130 (12.6)	
	2020 third quarter	117 (10.3)		382 (11.3)		865 (12.4)		6627 (26.8)	
	2020 fourth quarter	522 (46.1)		1670 (49.4)		3651 (52.4)		10,121 (40.9)	
	2021 first quarter	419 (37)		1139 (33.7)		2121 (30.4)		4642 (18.8)	
Family size, median (IQR)			2 (1)		2 (1)		2 (1)		2 (1)
**Regional division, n (%)^a^**									
	New England	50 (4.4)		129 (3.8)		252 (3.6)		1145 (4.6)	
	Mid-Atlantic	224 (19.8)		710 (21)		1140 (16.3)		4012 (16.2)	
	East North Central	56 (4.9)		220 (6.5)		690 (9.9)		2491 (10.1)	
	West North Central	91 (8)		246 (7.3)		625 (9)		1853 (7.5)	
	South Atlantic	296 (26.1)		856 (25.3)		1497 (21.5)		6740 (27.2)	
	East South Central	19 (1.7)		59 (1.7)		193 (2.8)		556 (2.2)	
	West South Central	136 (12)		454 (13.4)		1123 (16.1)		3209 (13)	
	Mountain	157 (13.9)		497 (14.7)		1076 (15.4)		3008 (12.2)	
	Pacific	62 (5.5)		100 (3)		209 (3)		1015 (4.1)	
	Others	41 (3.6)		107 (3.2)		169 (2.4)		728 (2.9)	

^a^States included for the regional divisions are New England (Connecticut, Maine, Massachusetts, New Hampshire, Rhode Island, and Vermont); Mid-Atlantic (New Jersey, New York, and Pennsylvania); East North Central (Illinois, Indiana, Michigan, Ohio, and Wisconsin); West North Central (Iowa, Kansas, Minnesota, Missouri, Nebraska, North Dakota, and South Dakota); South Atlantic (Delaware, Florida, Georgia, Maryland, North Carolina, South Carolina, Virginia, Washington, DC, and West Virginia); East South Central (Alabama, Kentucky, Mississippi, and Tennessee); West South Central (Arkansas, Louisiana, Oklahoma, and Texas); Mountain (Arizona, Colorado, Idaho, Montana, Nevada, New Mexico, Utah, and Wyoming); Pacific (Alaska, California, Hawaii, Oregon, and Washington).

**Table 2:** Associations between index age groups and SARS-CoV-2 transmission to household contacts.

Variables^a^		Index cases			Odds ratios (95% CI)		
		Associated with no secondary cases	Associated with any secondary cases	Crude model		Model 1	Model 2
**Age (years), n (%)**							
	0-4	934 (3)	198 (3.9)	1.45 (1.23-1.69)		1.69 (1.42-2.01)	1.69 (1.42-2.00)
	5-11	2797 (9)	581 (11.5)	1.42 (1.29-1.56)		1.30 (1.17-1.45)	1.29 (1.16-1.44)
	12-17	5849 (18.8)	1125 (22.2)	1.31 (1.22-1.41)		1.08 (0.99-1.17)	1.04 (0.96-1.13)
	18-25	21,597 (69.3)	3160 (62.4)	1 [Reference]		1 [Reference]	1 [Reference]
**Sex, n (%)**							
	Female	16,461 (52.8)	2603 (51.4)	—^b^		1 [Reference]	1 [Reference]
	Male	14,716 (47.2)	2461 (48.6)	—		0.99 (0.93-1.06)	0.99 (0.93-1.06)
**Quarter of first SARS-CoV-2 positive, n (%)**							
	2020 first quarter	234 (0.8)	23 (0.5)	—		0.94 (0.58-1.45)	0.99 (0.61-1.53)
	2020 second quarter	3177 (10.2)	531 (10.5)	—		0.81 (0.72-0.90)	0.80 (0.71-0.90)
	2020 third quarter	7250 (23.3)	741 (14.6)	—		0.57 (0.52-0.63)	0.57 (0.52-0.63)
	2020 fourth quarter	13,322 (42.7)	2642 (52.2)	—		1 [Reference]	1 [Reference]
	2021 first quarter	7194 (23.1)	1127 (22.3)	—		0.84 (0.78-0.92)	0.89 (0.82-0.97)
Family size, median (IQR)		2 (1)	2 (1)	—		2.63 (2.55-2.71)	2.66 (2.58-2.74)
**Regional divisions^c^**							
	New England	1370 (4.4)	206 (4.1)	—		—	1 [Reference]
	Mid-Atlantic	5259 (16.9)	827 (16.3)	—		—	1.32 (1.10-1.59)
	East North Central	2928 (9.4)	529 (10.4)	—		—	1.71 (1.41-2.08)
	West North Central	2418 (7.8)	397 (7.8)	—		—	1.83 (1.50-2.25)
	South Atlantic	8231 (26.4)	1158 (22.9)	—		—	1.34 (1.12-1.60)
	East South Central	720 (2.3)	107 (2.1)	—		—	1.55 (1.17-2.04)
	West South Central	4280 (13.7)	642 (12.7)	—		—	1.67 (1.39-2.02)
	Mountain	3880 (12.4)	858 (16.9)	—		—	1.80 (1.50-2.17)
	Pacific	1192 (3.8)	194 (3.8)	—		—	1.48 (1.17-1.87)
	Others	899 (2.9)	146 (2.9)	—		—	1.62 (1.25-2.08)

^a^Variables adjusted in the model are characteristics of the index cases.

^b^Indicates that only the age variable is included in the crude model and regional division is not included in model 1.

^c^States included for the regional divisions are New England (Connecticut, Maine, Massachusetts, New Hampshire, Rhode Island, and Vermont); Mid-Atlantic (New Jersey, New York, and Pennsylvania); East North Central (Illinois, Indiana, Michigan, Ohio, and Wisconsin); West North Central (Iowa, Kansas, Minnesota, Missouri, Nebraska, North Dakota, and South Dakota); South Atlantic (Delaware, Florida, Georgia, Maryland, North Carolina, South Carolina, Virginia, Washington, DC, and West Virginia); East South Central (Alabama, Kentucky, Mississippi, and Tennessee); West South Central (Arkansas, Louisiana, Oklahoma, and Texas); Mountain (Arizona, Colorado, Idaho, Montana, Nevada, New Mexico, Utah, and Wyoming); Pacific (Alaska, California, Hawaii, Oregon, and Washington).

## Discussion

### Principal Findings

In this study, we leveraged a large deidentified claims database to investigate the COVID-19 pandemic in the United States from January 2020 to March 2021. We identified valid households in the data and estimated the household transmission of SARS-CoV-2 by pediatric to young adult index cases aged 0 to 25 years. We calculated the overall SAR as 23.04% (95% CI 21.88-24.19). In a separate analysis of the same period, we found the overall SAR for all index ages (0 to 65+ years) to be 32.4% (95% CI 32.1-32.8). We observed infection peaks in April and December 2020. In the stratified analysis with pediatric index case age groups, we observed an inverse linear trend between the age groups and SARs—the oldest index case age group (18 to 25 years) had the lowest household transmission, which was statistically significant compared with the youngest group (0 to 4 years). When we analyzed the odds of household transmission of SARS-CoV-2 with a logistic multiple regression model, we obtained results similar to those found in the SAR analysis. Using the pediatric age group 18 to 25 years as the reference, we found that household transmission of SARS-CoV-2 in the 0 to 4 years age group was 1.69 times the odds of that in the reference group (95% CI 1.42-2.00). The differences between these 2 comparison groups were statistically significant across the 3 investigated models.

### Comparison to Prior Work

We found that pediatric-aged individuals did not make up the majority of the household index cases in the early waves of the COVID-19 pandemic. Only 10.9% (11,484/105,672) had pediatric index cases (age 0 to 17 years), which increased to 34% (36,241/105,672) if we included individuals aged 0 to 25 years. Similar proportions were also reported by others worldwide. A United States study reported that 14% (14/101) of the households investigated had pediatric index cases [12], while studies in Asia generally reported a lower percentage of pediatric index cases (age 0 to 20 years) in infected households: between 1% and 5% in China [17,18] and 3% in South Korea [19]. In Europe, the proportions were more comparable to those found in the United States: Denmark reported 5% (index age 0 to 20 years [20]); Greece reported 9% (index age 0 to 18 years [21]), and Switzerland reported 8%, (index age 0 to 16 years [22]). A comprehensive meta-analysis study suggested that the proportion was between 3% and 19% [23]. We emphasize that the SAR of 32.4% estimated for all age groups (0 to 65+ years) was larger than the overall SAR for the 0-to-25-year-old population. Additionally, it was more than 10% higher than the SAR for the young adult (18 to 25 years) population.

The reported household transmission of SARS-CoV-2 by pediatric and young adult index cases in the first year of the pandemic varied. In the United States, SARs in Tennessee and Wisconsin were 53% (95% CI 31-47) and 38% (95% CI 23-56) for index cases aged 0 to 12 years and 12 to 17 years, respectively [12]. The SAR was even higher in Los Angeles, California, at 81.9% (95% CI 72.1-91.9) for index children aged 0 to 18 years [24], which we speculate could be due to the inclusion of households with multiple index cases in the study. In a Denmark study, the SARs for young index case groups were lower, at 19% for the age group 0 to 10 years and 20% for the age group 10 to 20 years). Moreover, the ORs of viral transmission increased significantly as the age of the children index cases decreased [5]: age 0 to 5 years had an OR of 2.17 (95% CI 1.87-2.51), age 5 to 10 years had an OR of 1.66 (95% CI 1.50-1.83), and age 10 to 15 years had an OR of 1.37 (95% CI 1.26-1.49), with the reference age group being 15 to 20 years. Similar inverse relationships between the age of the pediatric index and SARS-CoV-2 transmission were reported in Norway [25], where the SARs were as follows: age 0 to 6 years, 24% (95% CI 20-28); age 7 to 12 years, 14% (95% CI 12-15); age 13 to 16 years, 14% (95% CI 13-16); and age 17 to 20 years, 11% (95% CI 10-13). Meanwhile, in Canada, the ORs were as follows: age 0 to 3 years, 1.43 (95% CI 1.17-1.75); age 4 to 8 years, 1.4 (95% CI 1.18-1.67); and age 9 to 13 years, 1.13 (95% CI 0.97-1.32), with the reference age group being 14 to 17 years.

However, this relationship was not evident in a South Korean investigation [19], where SARs were reported as 5.3% for ages 0 to 9 years (95% CI 1.3-13.7) and 18.6% for ages 10 to 19 years (95% CI 14-24). Upon further investigation, we posit that the SAR in South Korea for the age group spanning 0 to 9 years may not be reliable as it was calculated based on only 3 secondary cases (3/57). Recently, a meta-analysis of 45 studies showed that there was no significant difference in SARs between the age 0 to 19 years and age 20+ years index cases [23].

We found greater household infectivity of SARS-CoV-2 by the youngest index cases relative to their older counterparts (18 to 25 years) in this study. Although viral load is correlated with the spread of COVID-19 [26], several studies have reported that the amount of viral RNA in children is similar, or even higher, than that measured in adults [5,27,28]. Children are generally more likely to be asymptomatic after infection than adults [21,29], and asymptomatic cases are less capable of infecting susceptible individuals than symptomatic cases [18,23,30]. These observations contradict the results from our study and others [5,13,25]. Behavioral factors could play a bigger role in household SARS-CoV-2 transmission. Younger children require frequent and close contact care from their parents, and it may be difficult to quarantine children alone. Together, these factors could increase the infectivity of young children to parents and caregivers significantly when compared to the older, more independent, and self-caring index age group, such as those aged 18 to 25 years.

SARS-CoV-2 transmission is influenced by many factors, such as individuals’ demographic profiles and geographic locations [31]. Our regression analysis showed strong temporal and regional variations of the viral transmission in the United States SARs were high and over 30% in March and April 2020. It remained below 20% from May and September and rose to a peak value of approximately 30% in December 2020. We believe that these patterns could be explained partially by a few key events. Initially, the public was not prepared for the SARS-CoV-2, which caused high SARs. Later, national efforts were engaged, including state-level lockdowns and mask-wearing guidelines, to mitigate the risk of SARS-CoV-2 infection and transmission in the community [32]. From September onward to February 2021, the SAR pattern resembled that of the 7-day average of the national new cases [3]. During this period, changes in temperature, an increase in social activities related to the presidential election, and the availability of COVID-19 vaccines could be important factors explaining the observed trends. Among all the United States regions, New England had the lowest household transmission. This could be attributed to its high level of educational attainment, which also generally correlates with socioeconomic status [33].

Household size is a proxy for the number of susceptible contacts in estimating viral transmission. The association between household size and household transmission of SARS-CoV-2 is not consistent in the literature. Like our results, a study conducted in Canada reported a positive relationship (OR 1.63, 95% CI 1.43-1.86) [13]. However, investigations in the United States [12], England [4], and a meta-analysis of 6 international studies reported an inverse trend between the two [14]. Apart from the geographical and temporal factors, variations in the statistical modeling of the transmission outcome could also play a role in the discrepancy. Positive associations between household size and SARS-CoV-2 transmission were reported for studies defining the outcome as “any secondary cases in the household,” whereas a negative relationship was found in those studies modeling the outcome at the individual level.

In addition to the predictors investigated in our analysis, a wide range of factors are known to affect COVID-19 incidence. These include socioeconomic status, ethnicity, household composition, and numerous environmental factors like air pollution and meteorological conditions. For instance, the incidence rate ratios of the Social Vulnerability Index (SVI) to COVID-19 incidence and mortality were 1.14 (95% CI 1.13-1.16) and 1.14 (95% CI 1.12-1.16), respectively [34]. Similar findings were also observed for the subindexes of SVI. A meta-analysis also showed a weak correlation (0.36, 95% CI 0.02-0.62) between the average temperature and daily COVID-19 cases across cities from different countries [35].

### Strengths and Limitations

We acknowledge that there are 4 major limitations in our analysis of the administrative claims data. First, because we used data from a private health insurer, we were only able to include working individuals in the United States with insurance coverage. Additionally, adding to the potential bias, only a small proportion of valid households for estimating household SAR were filtered from the raw data (n=35,241, 0.85%; Figure 1). It may not be appropriate to compare the SARs obtained only from the excluded populations, such as families with self-employed and unemployed members, because there could be unique factors affecting SARs in those populations. Nevertheless, prior studies and our preliminary comparisons between the trends of national statistics estimated by claims data and those reported by the CDC are comparable [36,37]. Second, ethnicity is a risk factor for SARS-CoV-2 infection [38,39], and we were unable to account for its influence in estimating the SAR and modeling the household transmission in the logistic regression. Our estimations can only be interpreted as national averages and substantial differences between different ethnic groups are possible. Third, although we used a family identification variable to identify household members, we lacked the capability to fully identify multigenerational households (eg, with at least 1 adult aged 65 years or above, 1 adult in the working group aged 26 to 64 years, and 1 pediatric member aged 0 to 25 years). Commercial insurance subscribers are mostly working adults with spouses and children as dependents in their health plans. However, it is known that older adults (age 65 years and above) are more susceptible to SARS-CoV-2 infection and complications, and the SARs and regression estimates of transmission will likely only be larger and stronger than our study if such information is available. Finally, it is difficult to assess why individuals in a household might seek testing. One reason might be symptoms of COVID-19 among individuals; therefore, the rates will be biased toward estimating the SARs for symptomatic index cases.

Our study also has 2 strengths. First, we used data from the first year of the COVID-19 pandemic, and it is reasonable to assume that most of the contacts were not previously infected with SARS-CoV-2. Therefore, we can obtain an estimation of SARS-CoV-2 household transmission without information about prior history of infection or antibody test results. In addition, compared with the transmission estimations from meta-analysis, we used a single large cohort and were able to minimize the influence of analytic variation such as using a case definition, data filtering scheme, and model with the same set of predictors to estimate the range and heterogeneity of SAR across the time of the pandemic and various age groups of index cases.

### Conclusions

Our retrospective administrative claims study of viral transmission with 45,749 household contacts showed that large variations existed across months and regions during the initial waves of SARS-CoV-2 in the United States. The pediatric household SAR decreased from 32.0% to 21.1% as the age of the index cases increased and the SAR for all age groups was higher than the SAR for ages 0 to 25 years. There was also a positive association between family size and household transmission rate.

## Appendix

Multimedia Appendix 1 Graph and table of monthly household secondary attack rates of SARS-CoV-2 for pediatric index cases in the United States between March 2020 and February 2021.

Multimedia Appendix 2 Household secondary attack rates of SARS-CoV-2 stratified by the pediatric index age groups.

## Abbreviations

CDC: Centers for Disease Control and Prevention
LOINC: Logical Observation Identifiers Names and Codes
OR: odds ratio
PCR: polymerase chain reaction
SAR: secondary attack rate
SVI: Social Vulnerability Index

## Data Availability

In keeping with the user agreement with the data provider concerning the access to and use of the health research database, individual participant data used in this study and the data dictionary will not be available to the public. Researchers who are interested in the study and able to provide a scientifically sound proposal should contact the corresponding author (CJP) for collaboration. For reproducibility purposes, all analytic code will be provided upon request.
