# Supplementary material for: Pediatric and Young Adult Household Transmission of the Initial Waves of SARS-CoV-2 in the United States: Administrative Claims Study
Source: J Med Internet Res. 2024 Jan 4;26:e44249. doi: 10.2196/44249 (PMC10768807; doi:10.2196/44249)
Supplement: Multimedia Appendix 2 [file jmir_v26i1e44249_app2.docx]

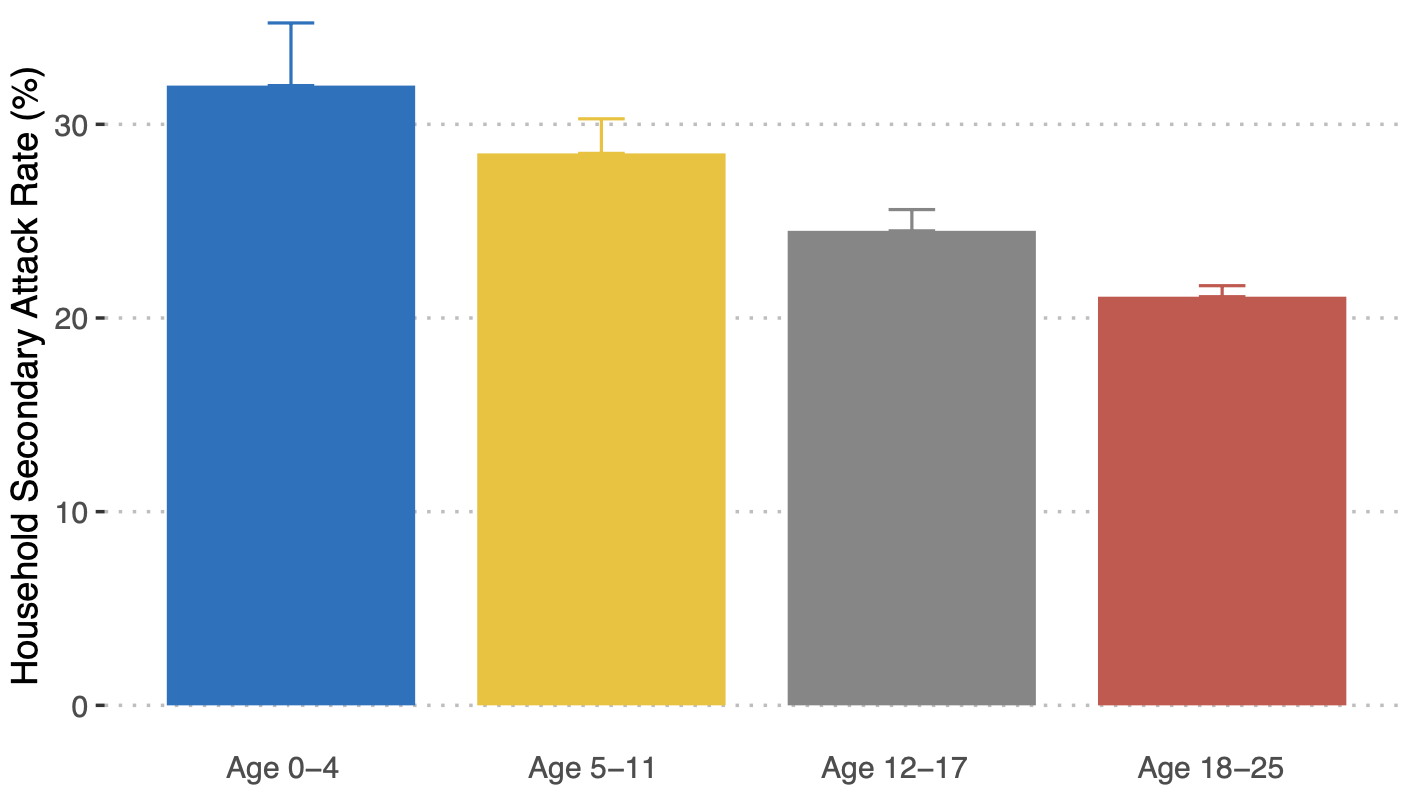


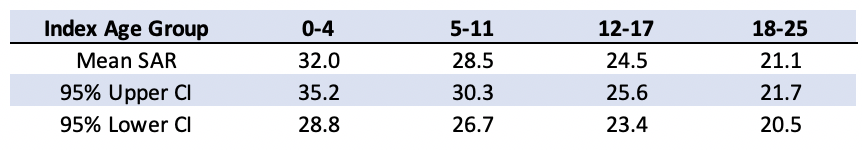


Figure S2. Household secondary attack rates of SARS-CoV-2 stratified by the pediatric index age groups. The SAR for all age groups was 32.4%.
